# Supplementary material for: Angiogenic and immune predictors of neoadjuvant axitinib response in renal cell carcinoma with venous tumour thrombus
Source: Nat Commun. 2025 Apr 28;16:3870. doi: 10.1038/s41467-025-58436-8 (PMC12037771; doi:10.1038/s41467-025-58436-8)
Supplement: Supplementary file 1 — Supplementary Information [file 41467_2025_58436_MOESM1_ESM.pdf]

Supplementary Information

Contents:

1. **Supplementary Figures S1-11**
2. **Supplementary Tables S1-5**

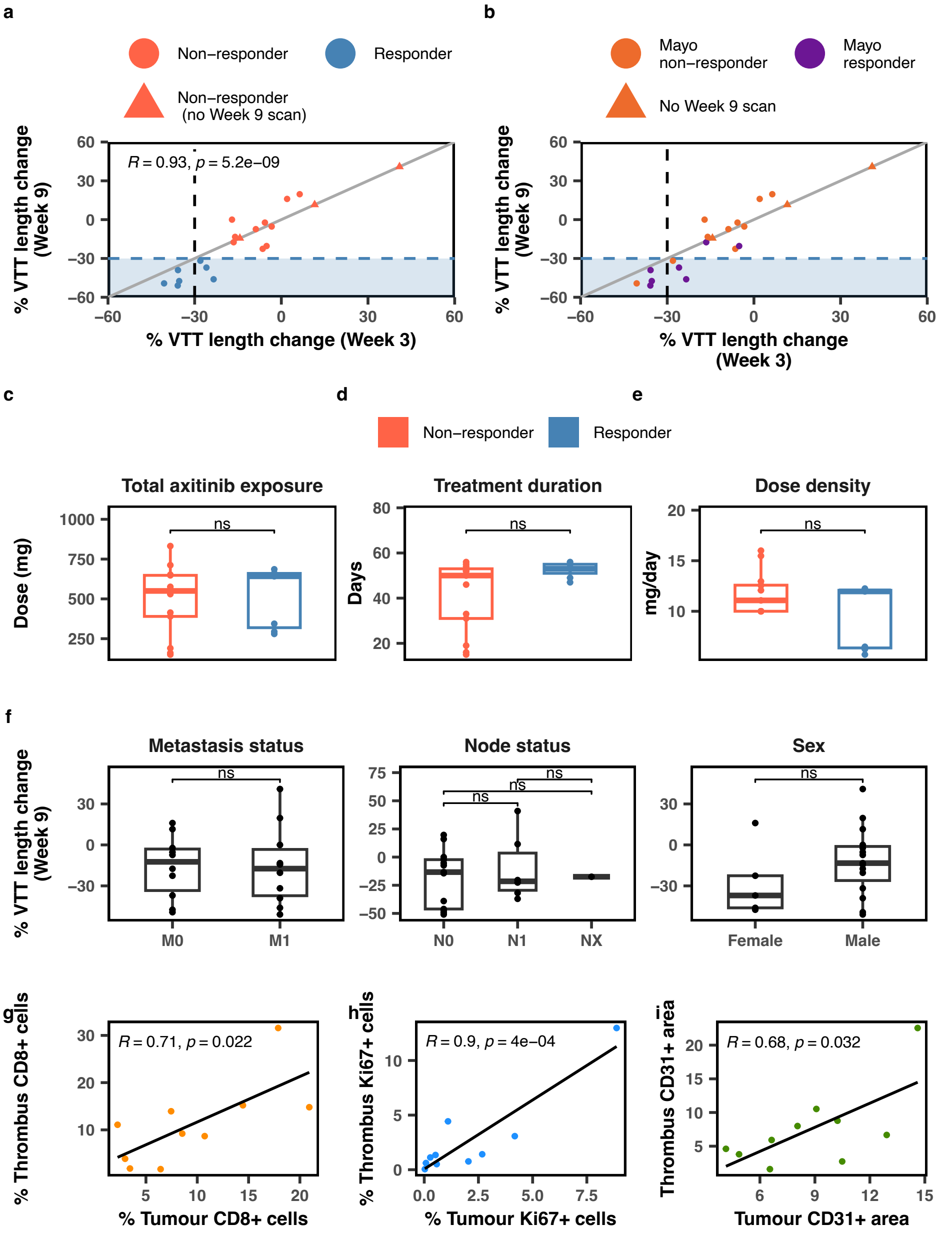

Figure S1. Treatment response as assessed by VTT length on the NAXIVA Trial
a, Association between MRI response at week 3 and week 9 (simple linear regression). Patients that stopped at week 3 are plotted along the line of week 3 = week 9 and were excluded from the correlation analysis. (n=7 responders, 10 non-responders and 3 stopped early). **b,** Association between MRI response at week 3 and week 9, with response annotated by Mayo classification. Note that two patients with good length response would be classified as Mayo non-responders. **c,** Total axitinib received on trial. (n=7 responders, 13 non-responders). **d,** Duration of drug treatment on trial for each patient. (n=7 responders, 13 non-responders). **e,** Dose density (total mg / number of days treatment) for each patient. (n=7 responders, 13 non-responders). **f,** VTT response by metastasis stage, lymph node stage and in male and female participants. (n=10 M0, 10 M1. n=13 N0, 6 N1 and 1 NX. n=5 females and 15 males). **g-i,** Whole IHC slides were quantitated by HALO analysis for Ki67 **(g),** CD8 **(h)** and CD31 **(i)** in n=10 untreated paired primary tumours and VTTs.
Pearson correlations between quantification of markers in VTT and the paired primary tumour are shown in **g-i**. For all other comparisons: unpaired Student's T-test with Bonferroni correction. All box plots show the median (centre line), upper and lower quartiles (box bounds) and whiskers extending to 1.5x interquartile range. ns: p>0.05, *: p≤0.05, **: p≤0.01, ***: p≤ 0.001]

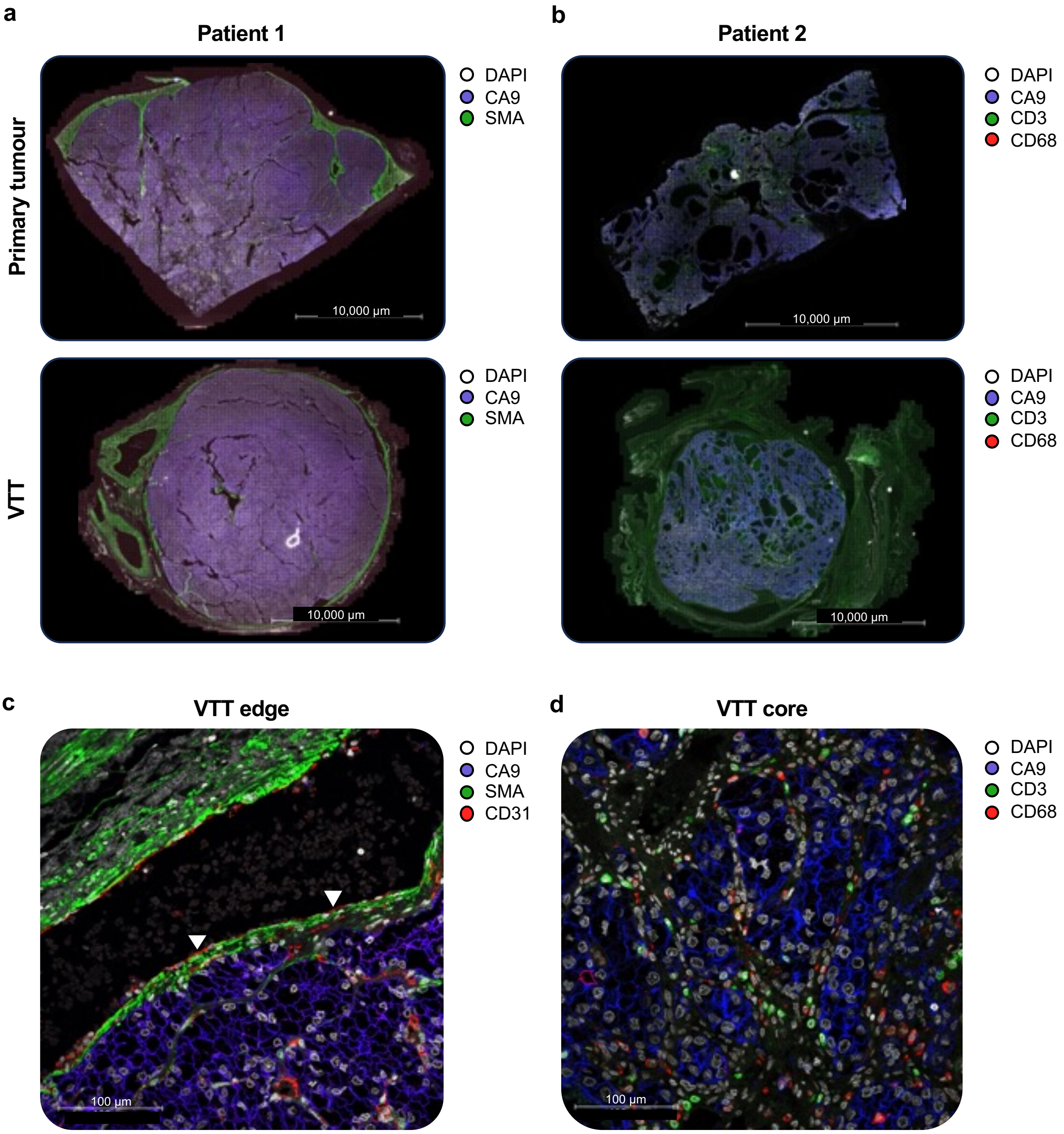

Figure S2. Untreated microenvironment of VTT.
Additional scans of tumour and VTT.
a-b, Paired primary tumour and VTT cases stained for (**a**) vascular and (**b**) immune markers. CA9+ viable tumour fills the lumen of the renal vein in both cases.
c-d, Higher magnification confocal images of the VTT. (**c**) The VTT edge shows CD31+ endothelial cells and SMA+ stromal cells on the surface of the VTT (white arrows). The adjacent SMA + normal vein is visible. (**d**) Immune cells are visible in the VTT core.

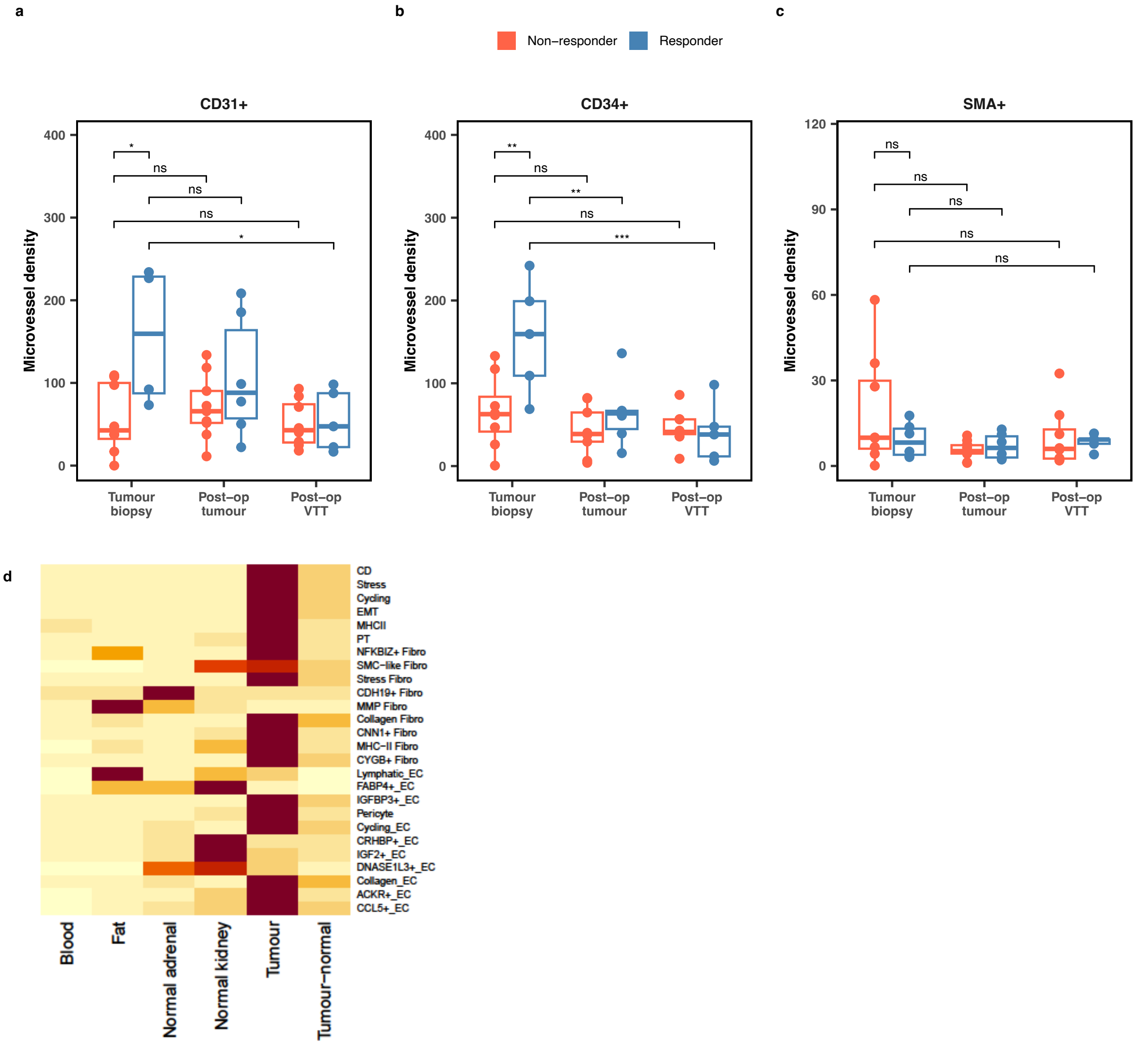

Figure S3. Angiogenesis

a, Change in CD31+ microvessel density before and after treatment. (p=0.0287 for responder to non-responder tumour biopsy comparison and p=0.0481 for responder biopsy to post-op VTT comparison; n=12 tumour biopsies [4 responders, 8 non-responders], 15 post-op tumour samples [6 responders, 9 non-responders], and 13 post-op VTT samples [5 responders, 8 non-responders]). **b,** Change in CD34+ microvessel density before and after treatment. ($p=4.27x10^{-3}$ for responder to non-responder tumour biopsy comparison, $p=7.21x10^{-3}$ for responder biopsy to post-op tumour comparison, and $p=8.02x10^{-4}$ for responder biopsy to post-op VTT comparison; n=13 tumour biopsies [5 responders, 8 non-responders], 15 post-op tumour samples [6 responders, 9 non-responders], 13 post-op VTT samples [5 responders, 8 non-responders]). **c,** Change in SMA+ CAF area coverage before and after treatment. (n=14 tumour biopsies [6 responders, 8 non-responders], 16 post-op tumour samples [6 responders, 10 non-responders], and 13 post-op VTT samples [5 responders, 8 non-responders]). ns: p>0.05, *: p≤0.05, **: p≤0.01, ***: p≤ 0.001. All box plots show the median (centre line), upper and lower quartiles (box bounds) and whiskers extending to 1.5x interquartile range. All statistical tests are one-way ANOVA with Tukey's post-hoc test.

d, Cell prevalence by region in single cell dataset.

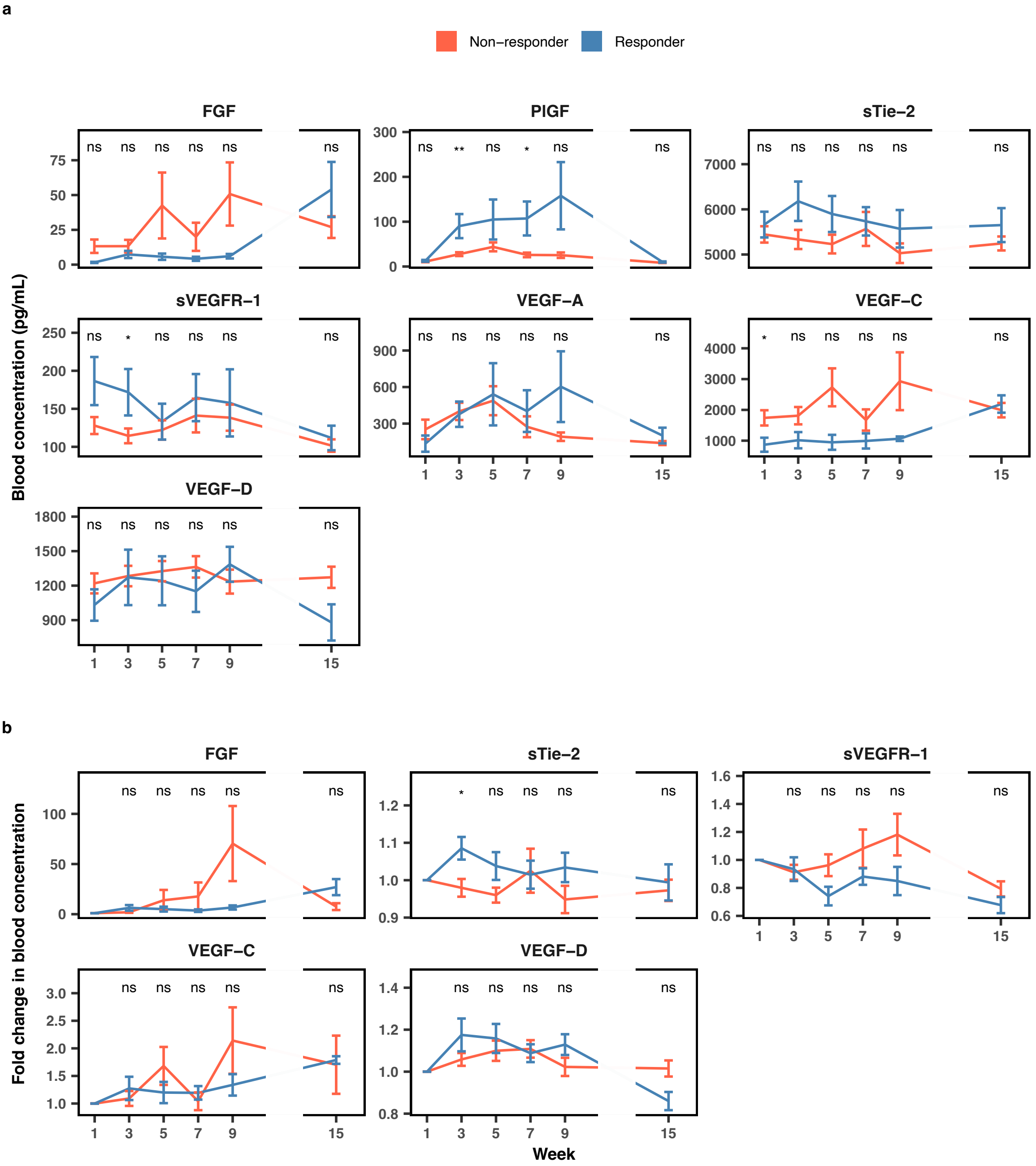

Figure S4. Angiogenic factors

a, Absolute and **b,** fold change measurements of angiogenic factors during the trial (unpaired Student's T-test ± SEM; p=8.19x10-3 for absolute PlGF week 3 comparison and 0.0169 for week 7, p=0.0356 for absolute VEGF-C week 1 comparison, p=0.0445 for sVEGFR-1 week 3 comparison and p=0.0165 for fold change sTie-2 week 3 comparison; n=19 for weeks 1-7 [7 responders, 12 non-responders], 14 for week 9 [5 responders, 9 non-responders], 11 for the post-op measurement [4 responders, 7 non-responders]). ns: p>0.05, *: p≤0.05, **: p≤0.01, ***: p≤ 0.001.

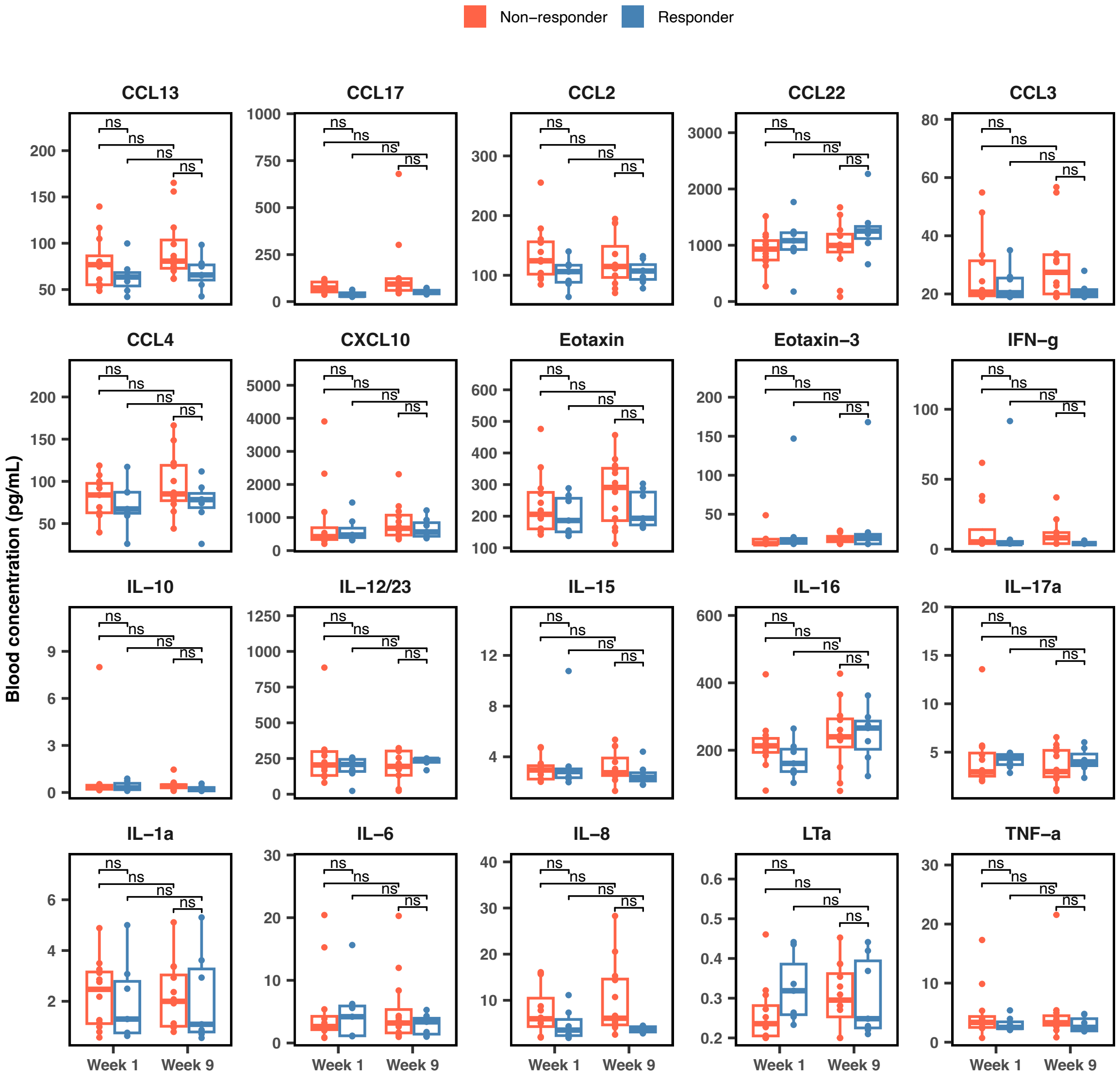

Figure S5. Extended plasma cytokines
Plasma cytokine levels were quantified at the beginning and end of axitinib treatment by cytokine array (one-way ANOVA with Tukey's post-hoc test; n=19 [7 responders, 12 non-responders]). All box plots show the median (centre line), upper and lower quartiles (box bounds) and whiskers extending to 1.5x interquartile range. ns: p>0.05, *: p≤0.05, **: p≤0.01, ***: p≤ 0.001.

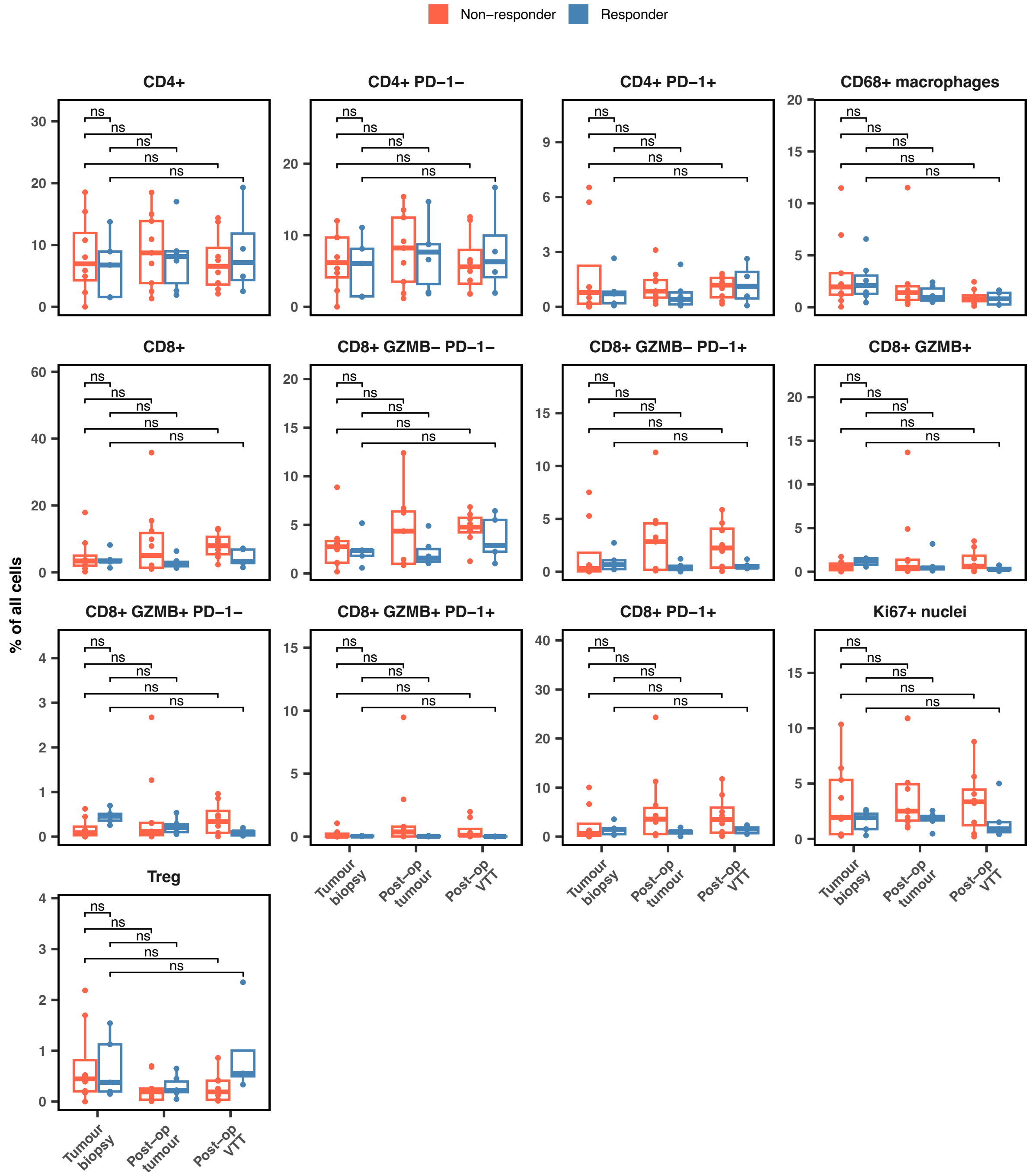

Figure S6. Tissue immune subsets

Multiplex immunofluorescence slides were quantified using HALO software. (One-way ANOVA with Tukey's post-hoc test; n=13 tumour biopsies [5 responders, 8 non-responders], 15 post-op tumour samples [6 responders, 9 non-responders], 12 post-op VTT samples [4 responders, 8 non-responders]). All box plots show the median (centre line), upper and lower quartiles (box bounds) and whiskers extending to 1.5x interquartile range. ns: p>0.05, *: p≤0.05, **: p≤0.01, ***: p≤ 0.001

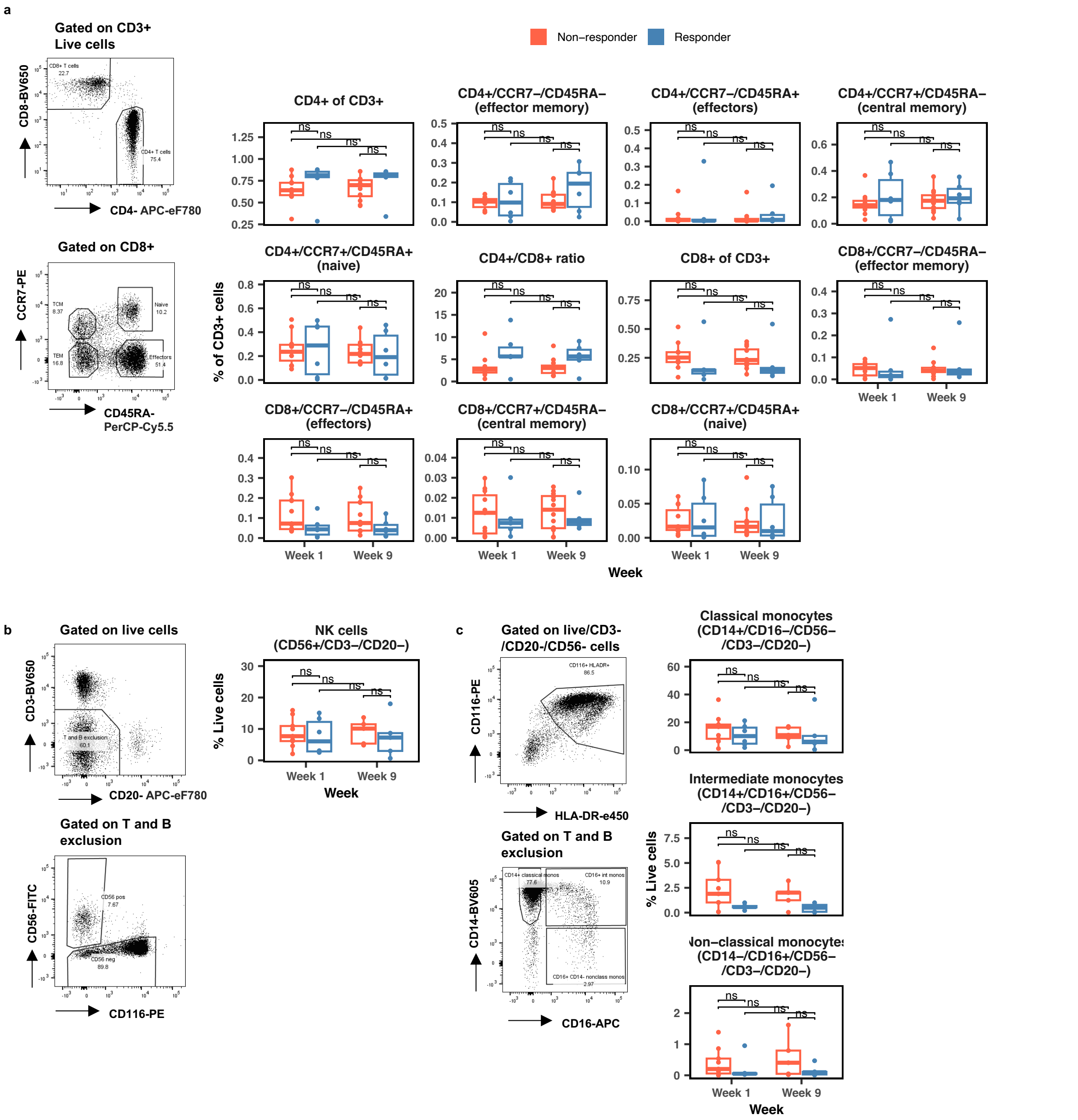

Figure S7. Peripheral immune cell profile

a, Representative image of gating strategy and CD4+ and CD8+ T cell subsets by CCR7 and CD45RA status, as percentage of CD3+/live cells (one-way ANOVA with Tukey's post-hoc test; n=17 at week 1 [6 responders, 11 non-responders], 18 at week 9 [6 responders, 12 non-responders]). **b-c,** Gating strategy & percentage of live cells for **b,** natural killer cells and **c,** monocyte subsets (unpaired Student's T-test with Bonferroni correction; n=15 at week 1 [6 responders, 9 non-responders], 10 at week 9 [5 responders, 5 non-responders]). All box plots show the median (centre line), upper and lower quartiles (box bounds) and whiskers extending to 1.5x interquartile range. ns: p>0.05, *: p≤0.05, **: p≤0.01, ***: p≤ 0.001.

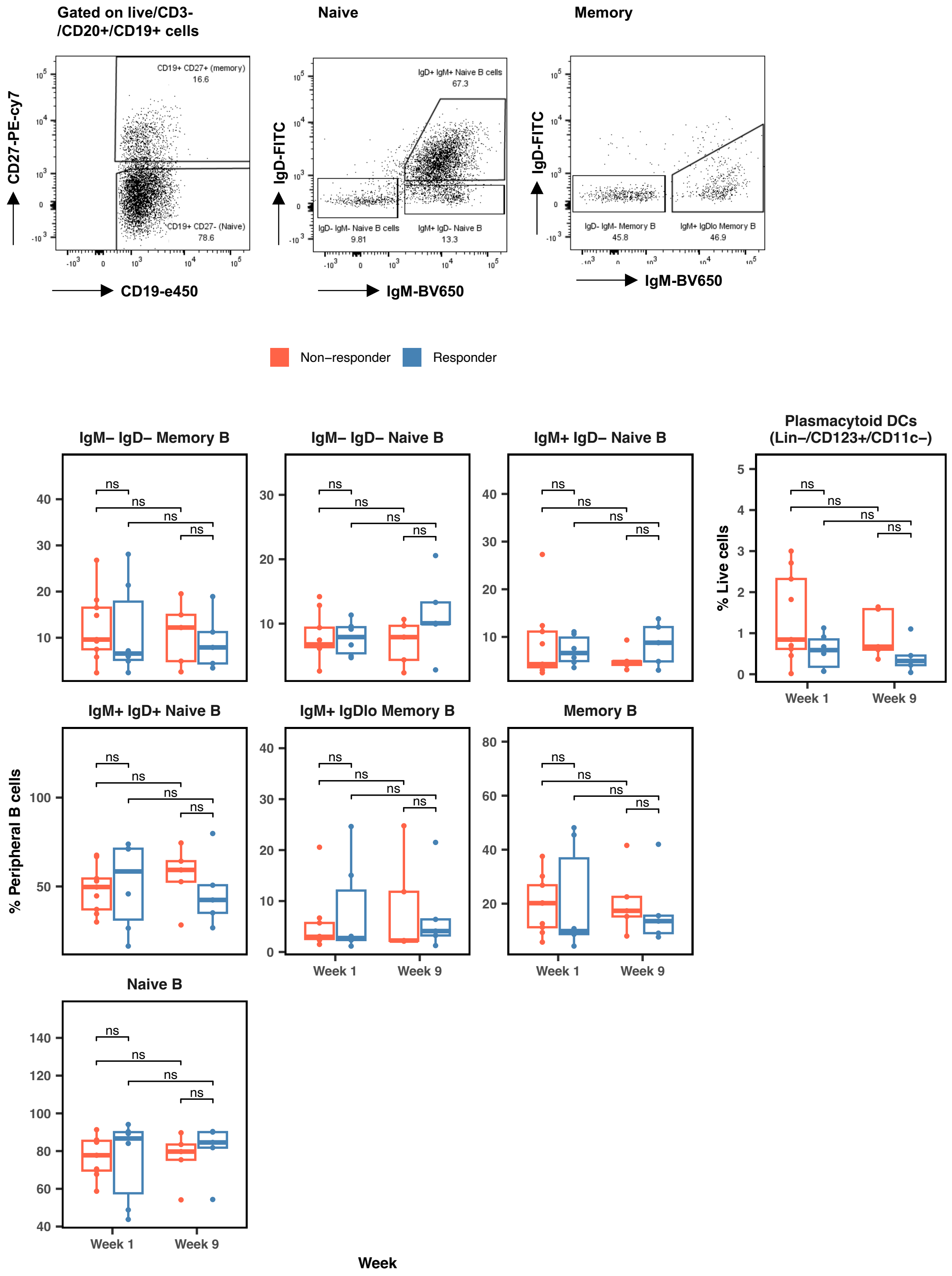

Figure S8. Blood immune profile

B cell subsets and plasmacytoid DCs from PBMCs taken at baseline and at the end of axitinib treatment were analysed by multicolour flow cytometry. B cell gating strategy is shown. (Unpaired Student's T-test with Bonferroni correction; n=15 at week 1 [6 responders, 9 non-responders], 10 at week 9 [5 responders, 5 non-responders]). All box plots show the median (centre line), upper and lower quartiles (box bounds) and whiskers extending to 1.5x interquartile range. ns: p>0.05, *: p≤0.05, **: p≤0.01, ***: p≤ 0.001.

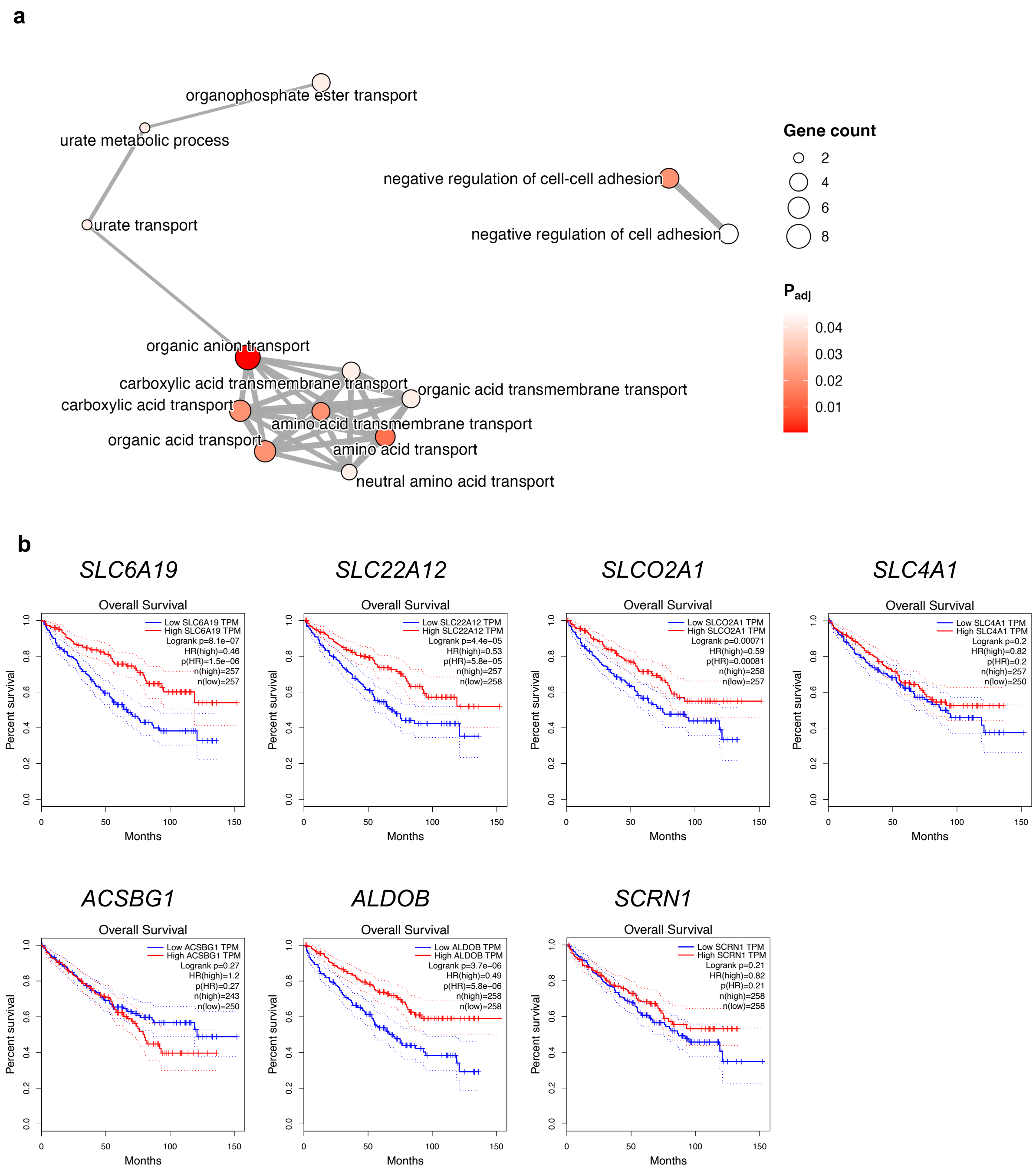

Figure S9. RNA-seq GO analysis results

a, GO analysis network for genes achieving P < 0.01 in the differential expression analysis. **b,** Survival plots of kidney cancer patients differentiated according to the expression of solute carrier genes highly expressed in responders to axitinib in NAXIVA.

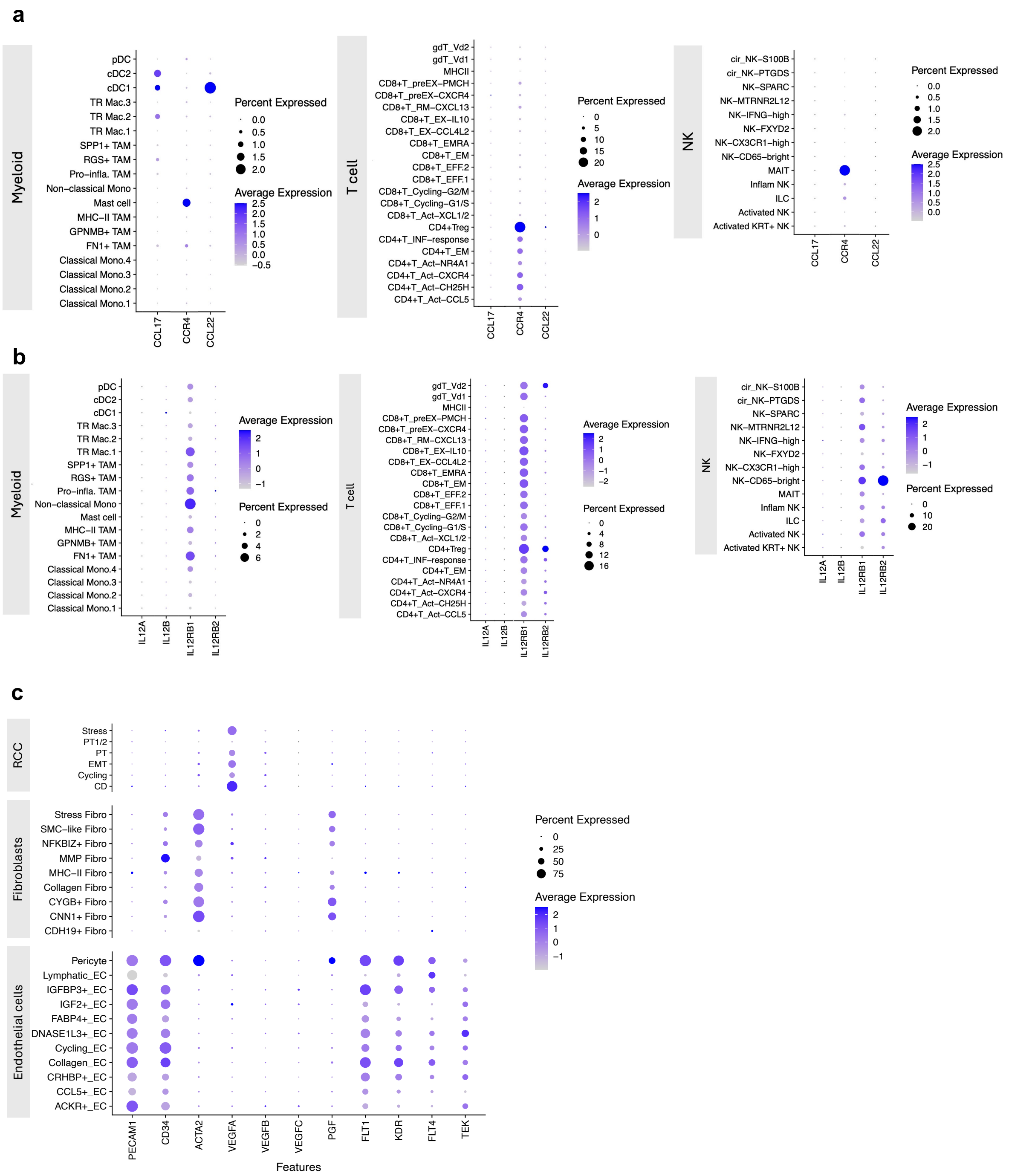

Figure S10. scRNA-seq compartments expressing key features

a, Cells expressing *CCL17*, its receptor *CCR4* and its antagonist *CCL22* according to single cell RNA-seq data. **b,** Cells expressing *IL12A*, *IL12B*, and the receptors *IL12RB1* and *IL12RB2* according to single cell RNA-seq data. **c,** Cells expressing angiogenesis-related features.

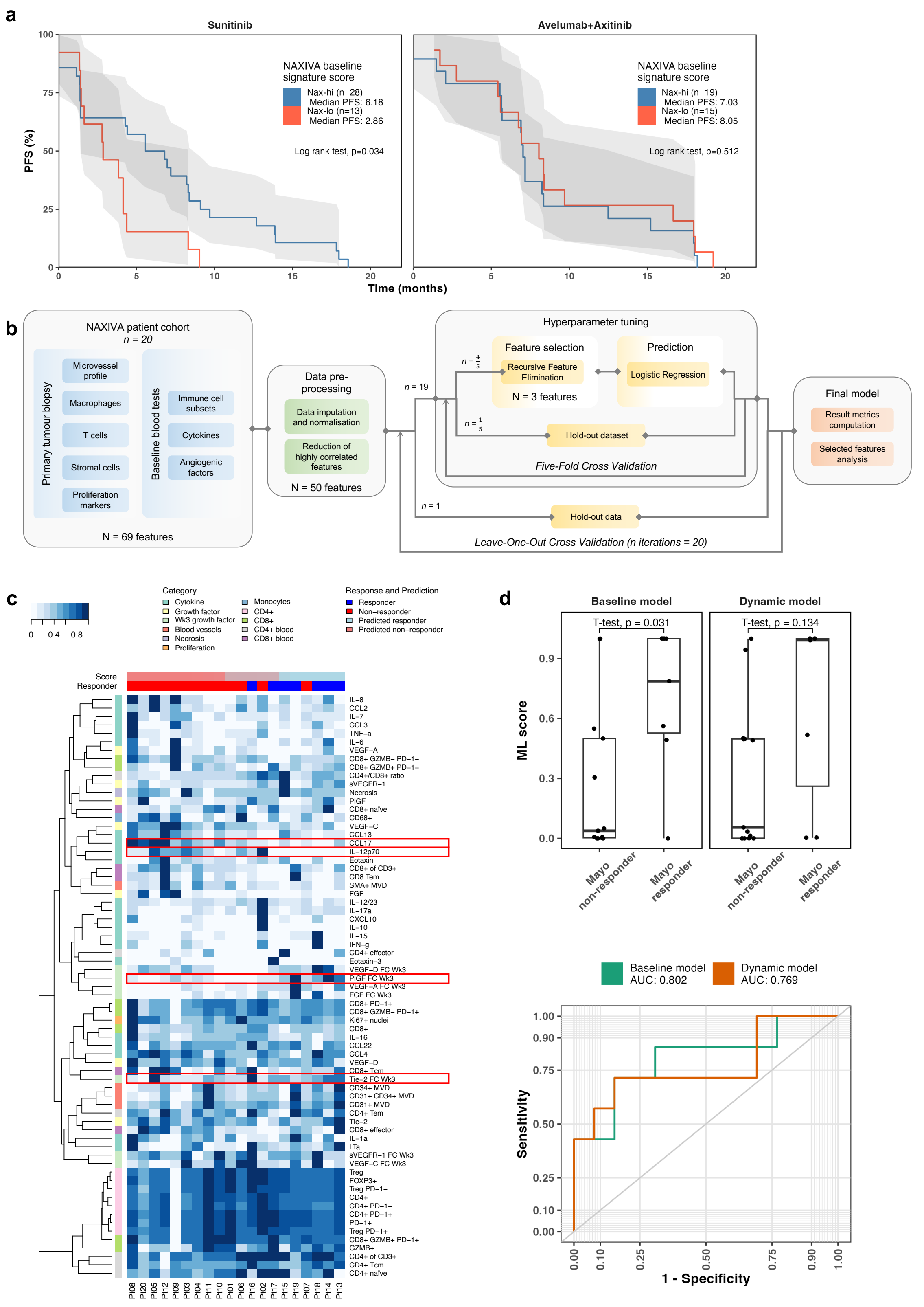

Figure S11. Machine learning features and model design.

a, The NAXIVA baseline signature can stratify patients in the sunitinib arm of the Javelin Renal 101 trial [15]. **b,** Schematic of Model 2. **c,** Panel plot for Model 2 highlighting highest selected features. **d,** ML model prediction of response according to Mayo classification. All box plots show the median (centre line), upper and lower quartiles (box bounds) and whiskers extending to 1.5x interquartile range.

Table S1. AUC and feature selection results for machine learning models with different number of features pre-specified for selection by the Recursive Feature Elimination (RFE) algorithm. Only the top N selected features are listed for every “N features”, based on their selection frequency. The approaches include:

- Selection from baseline features only (this approach with N=3 selected features was further analysed as the ‘baseline model’),
- Selection from baseline and dynamic week 3 features (this approach with N=3 selected features was analysed as the ‘dynamic model’),
- Selection from dynamic week 3 features, concatenated with a fixed set of 3 baseline features (CCL17, IL-12p70, and CD31+ MVD, manually pre-specified based on results from the baseline model).

N features	Baseline only	Baseline + Dynamic	Fixed Baseline + Model-selected Dynamic
2	AUC = 0.802<br>Baseline: CCL17, IL-12p70	AUC = 0.670<br>Baseline: CCL17<br>Dynamic: PIGF	N/A
3	AUC = 0.868<br>Baseline: CCL17, IL-12p70, CD31+ MVD	AUC = 0.945<br>Baseline: CCL17<br>Dynamic: PIGF, sTie-2	N/A
4	AUC = 0.758<br>Baseline: CCL17, IL-12p70, CD31+ MVD, sVEGFR-1	AUC = 0.593<br>Baseline: CCL17, IL-12p70<br>Dynamic: PIGF, sTie-2	AUC = 0.769<br>Baseline (fixed): CCL17, IL-12p70, CD31+ MVD<br>Dynamic: PIGF
5	AUC = 0.802<br>Baseline: CCL17, IL-12p70, CD31+ MVD, sVEGFR-1, LTa	AUC = 0.780<br>Baseline: CCL17, IL-12p70, sVEGFR-1<br>Dynamic: PIGF, sTie-2	AUC = 0.813<br>Baseline (fixed): CCL17, IL-12p70, CD31+ MVD<br>Dynamic: PIGF, sTie-2
6	AUC = 0.813<br>Baseline: CCL17, IL-12p70, CD31+ MVD, sVEGFR-1, LTa, FGF	AUC = 0.868<br>Baseline: CCL17, IL-12p70, sVEGFR-1, FGF<br>Dynamic: PIGF, sTie-2	AUC = 0.934<br>Baseline (fixed): CCL17, IL-12p70, CD31+ MVD<br>Dynamic: PIGF, sTie-2, FGF
7	AUC = 0.714<br>Baseline: CCL17, IL-12p70, CD31+ MVD, sVEGFR-1, LTa, FGF, IL-7	AUC = 0.8791<br>Baseline: CCL17, IL-12p70, sVEGFR-1, FGF<br>Dynamic: PIGF, sTie-2, FGF	AUC = 0.934<br>Baseline (fixed): CCL17, IL-12p70, CD31+ MVD<br>Dynamic: PIGF, sTie-2, FGF, VEGF-A

Table S2. Flow cytometry panels

Panel	Marker	Fluorophore	Manufacturer	Catalogue Number	Clone
B cell	IgM	BV650	BioLegend	314526	MHM-88
B cell	IgD	FITC	BD	555778	IA6-2
B cell	CD27	PE-Cyanine7	ThermoFisher	25-0279-42	O323
B cell	CD19	V450	BD	560353	HIB19
Myeloid/NK	CD16	APC	ThermoFisher	17-0168-42	eBioCB16
Myeloid/NK	CD19	APC-eFluor780	ThermoFisher	47-0199-42	HIB19
Myeloid/NK	CD20	APC-eFluor780	ThermoFisher	47-0209-42	2H7
Myeloid/NK	CD14	BV605	BioLegend	301834	M5E2
Myeloid/NK	CD3	BV650	BioLegend	317324	OKT3
Myeloid/NK	HLA-DR	eFluor450	ThermoFisher	48-9952-42	L243
Myeloid/NK	CD56	FITC	ThermoFisher	11-0566-42	TULY56
Myeloid/NK	CD116	PE	BD	551373	hGMCSFR-M1
Myeloid/NK	CD11c	PE-Vio770	Miltenyi	130-113-581	MJ4-27G12
Myeloid/NK	CD123	PerCP-Cyanine5.5	ThermoFisher	45-1239-42	6H6
T cell	CD4	APC-eFluor780	ThermoFisher	47-0049-42	RPA-T4
T cell	CD8a	BV650	BioLegend	301042	RPA-T8
T cell	CCR7	PE	BioLegend	353204	G043H7
T cell	CD45RA	PerCP-Cyanine5.5	ThermoFisher	45-0458-42	HI100
T cell, B cell	CD3	BV605	BioLegend	317322	OKT3
T cell, B cell, Myeloid/NK	Viability	Zombie AQUA	BioLegend	423101	

Table S3. Full list of features for machine learning models.

Measurement	Timepoint
IFN-g	baseline
IL-10	baseline
IL-12p70	baseline
IL-6	baseline
IL-8	baseline
TNF-a	baseline
Eotaxin	baseline
Eotaxin-3	baseline
CXCL10	baseline
CCL2	baseline
CCL13	baseline
CCL22	baseline
CCL3	baseline
CCL4	baseline
CCL17	baseline
IL-1a	baseline
IL-12/23	baseline
IL-15	baseline
IL-16	baseline
IL-17a	baseline
IL-7	baseline
LTa	baseline
FGF	baseline
sVEGFR-1	baseline
PlGF	baseline
Tie-2	baseline
VEGF-A	baseline
VEGF-C	baseline
VEGF-D	baseline
FGF	week_3
sVEGFR-1	week_3
PlGF	week_3
Tie-2	week_3
VEGF-A	week_3
VEGF-C	week_3
VEGF-D	week_3
Necrosis	baseline
CD31+ area	baseline
CD34+ area	baseline
CD31+ CD34+ area	baseline
CD68+	baseline
SMA+ area	baseline
Ki67+ nuclei	baseline
CD8+	baseline
GZMB+	baseline
CD8+ PD-1+	baseline
CD8+ GZMB+ PD-1-	baseline
CD8+ GZMB- PD-1+	baseline
CD8+ GZMB+ PD-1+	baseline
CD8+ GZMB- PD-1-	baseline
CD4+	baseline
FOXP3+	baseline
PD-1+	baseline
Treg	baseline
Treg PD-1+	baseline
Treg PD-1-	baseline
CD4+ PD-1+	baseline
CD4+ PD-1-	baseline
CD4+ of CD3+	baseline
CD8+ of CD3+	baseline
CD4+/CD8+ ratio	baseline
CD4+ effector	baseline
CD4+ naïve	baseline
CD4+ Tcm	baseline
CD4+ Tem	baseline
CD8+ effector	baseline
CD8+ naïve	baseline
CD8+ Tcm	baseline
CD8 Tem	baseline

Table S4. Hyperparameter ranges

Hyperparameter	Range
alpha	[0.0001, 0.001, 0.01, 0.1, 1, 10, 100]
penalty	['l1', 'l2', 'elasticnet']
learning_rate	['optimal', 'invscaling', 'adaptive']
eta0	[0.01, 0.1, 1]

Table S5. Selected hyperparameters for each leave-one-out iteration of the model.

Model	Method	Algorithm	Iteration	Hyperparameters
Baseline	Logistic Regression RFE	Logistic SGD	0	[{'alpha': 0.0001, 'eta0': 0.01, 'learning_rate': 'optimal', 'penalty': 'l1'}]
Baseline	Logistic Regression RFE	Logistic SGD	1	[{'alpha': 0.0001, 'eta0': 0.01, 'learning_rate': 'optimal', 'penalty': 'l1'}]
Baseline	Logistic Regression RFE	Logistic SGD	2	[{'alpha': 0.0001, 'eta0': 0.01, 'learning_rate': 'optimal', 'penalty': 'l1'}]
Baseline	Logistic Regression RFE	Logistic SGD	3	[{'alpha': 0.0001, 'eta0': 0.01, 'learning_rate': 'optimal', 'penalty': 'l1'}]
Baseline	Logistic Regression RFE	Logistic SGD	4	[{'alpha': 0.0001, 'eta0': 0.01, 'learning_rate': 'optimal', 'penalty': 'l1'}]
Baseline	Logistic Regression RFE	Logistic SGD	5	[{'alpha': 0.0001, 'eta0': 0.01, 'learning_rate': 'optimal', 'penalty': 'l1'}]
Baseline	Logistic Regression RFE	Logistic SGD	6	[{'alpha': 0.0001, 'eta0': 0.01, 'learning_rate': 'optimal', 'penalty': 'l1'}]
Baseline	Logistic Regression RFE	Logistic SGD	7	[{'alpha': 0.0001, 'eta0': 0.01, 'learning_rate': 'optimal', 'penalty': 'l1'}]
Baseline	Logistic Regression RFE	Logistic SGD	8	[{'alpha': 0.0001, 'eta0': 0.01, 'learning_rate': 'optimal', 'penalty': 'l1'}]
Baseline	Logistic Regression RFE	Logistic SGD	9	[{'alpha': 0.0001, 'eta0': 0.01, 'learning_rate': 'optimal', 'penalty': 'l1'}]
Baseline	Logistic Regression RFE	Logistic SGD	10	[{'alpha': 0.0001, 'eta0': 0.01, 'learning_rate': 'optimal', 'penalty': 'l1'}]
Baseline	Logistic Regression RFE	Logistic SGD	11	[{'alpha': 0.0001, 'eta0': 0.01, 'learning_rate': 'optimal', 'penalty': 'l1'}]
Baseline	Logistic Regression RFE	Logistic SGD	12	[{'alpha': 0.0001, 'eta0': 0.01, 'learning_rate': 'optimal', 'penalty': 'l1'}]
Baseline	Logistic Regression RFE	Logistic SGD	13	[{'alpha': 0.0001, 'eta0': 0.01, 'learning_rate': 'optimal', 'penalty': 'l1'}]
Baseline	Logistic Regression RFE	Logistic SGD	14	[{'alpha': 100, 'eta0': 0.1, 'learning_rate': 'invscaling', 'penalty': 'l2'}]
Baseline	Logistic Regression RFE	Logistic SGD	15	[{'alpha': 0.0001, 'eta0': 0.01, 'learning_rate': 'optimal', 'penalty': 'l1'}]
Baseline	Logistic Regression RFE	Logistic SGD	16	[{'alpha': 100, 'eta0': 0.1, 'learning_rate': 'invscaling', 'penalty': 'l2'}]
Baseline	Logistic Regression RFE	Logistic SGD	17	[{'alpha': 0.0001, 'eta0': 0.01, 'learning_rate': 'optimal', 'penalty': 'elasticnet'}]
Baseline	Logistic Regression RFE	Logistic SGD	18	[{'alpha': 0.0001, 'eta0': 0.01, 'learning_rate': 'optimal', 'penalty': 'l1'}]
Baseline	Logistic Regression RFE	Logistic SGD	19	[{'alpha': 0.0001, 'eta0': 0.01, 'learning_rate': 'optimal', 'penalty': 'l1'}]
Dynamic	Logistic Regression RFE	Logistic SGD	0	[{'alpha': 0.001, 'eta0': 0.01, 'learning_rate': 'optimal', 'penalty': 'elasticnet'}]
Dynamic	Logistic Regression RFE	Logistic SGD	1	[{'alpha': 0.0001, 'eta0': 0.01, 'learning_rate': 'invscaling', 'penalty': 'l2'}]
Dynamic	Logistic Regression RFE	Logistic SGD	2	[{'alpha': 0.0001, 'eta0': 0.1, 'learning_rate': 'optimal', 'penalty': 'l1'}]
Dynamic	Logistic Regression RFE	Logistic SGD	3	[{'alpha': 100, 'eta0': 0.1, 'learning_rate': 'invscaling', 'penalty': 'l2'}]
Dynamic	Logistic Regression RFE	Logistic SGD	4	[{'alpha': 0.0001, 'eta0': 0.01, 'learning_rate': 'optimal', 'penalty': 'l1'}]
Dynamic	Logistic Regression RFE	Logistic SGD	5	[{'alpha': 0.001, 'eta0': 0.01, 'learning_rate': 'optimal', 'penalty': 'l2'}]
Dynamic	Logistic Regression RFE	Logistic SGD	6	[{'alpha': 0.0001, 'eta0': 0.01, 'learning_rate': 'optimal', 'penalty': 'l1'}]
Dynamic	Logistic Regression RFE	Logistic SGD	7	[{'alpha': 0.0001, 'eta0': 0.01, 'learning_rate': 'optimal', 'penalty': 'l1'}]
Dynamic	Logistic Regression RFE	Logistic SGD	8	[{'alpha': 0.0001, 'eta0': 0.01, 'learning_rate': 'optimal', 'penalty': 'elasticnet'}]
Dynamic	Logistic Regression RFE	Logistic SGD	9	[{'alpha': 10, 'eta0': 1, 'learning_rate': 'invscaling', 'penalty': 'l2'}]
Dynamic	Logistic Regression RFE	Logistic SGD	10	[{'alpha': 0.0001, 'eta0': 0.01, 'learning_rate': 'optimal', 'penalty': 'elasticnet'}]
Dynamic	Logistic Regression RFE	Logistic SGD	11	[{'alpha': 0.0001, 'eta0': 0.01, 'learning_rate': 'invscaling', 'penalty': 'l2'}]
Dynamic	Logistic Regression RFE	Logistic SGD	12	[{'alpha': 0.0001, 'eta0': 0.01, 'learning_rate': 'optimal', 'penalty': 'l1'}]
Dynamic	Logistic Regression RFE	Logistic SGD	13	[{'alpha': 0.0001, 'eta0': 1, 'learning_rate': 'optimal', 'penalty': 'elasticnet'}]
Dynamic	Logistic Regression RFE	Logistic SGD	14	[{'alpha': 0.0001, 'eta0': 0.01, 'learning_rate': 'optimal', 'penalty': 'l2'}]
Dynamic	Logistic Regression RFE	Logistic SGD	15	[{'alpha': 0.0001, 'eta0': 0.01, 'learning_rate': 'optimal', 'penalty': 'l1'}]
Dynamic	Logistic Regression RFE	Logistic SGD	16	[{'alpha': 0.0001, 'eta0': 0.01, 'learning_rate': 'optimal', 'penalty': 'l1'}]
Dynamic	Logistic Regression RFE	Logistic SGD	17	[{'alpha': 0.0001, 'eta0': 0.01, 'learning_rate': 'optimal', 'penalty': 'l1'}]
Dynamic	Logistic Regression RFE	Logistic SGD	18	[{'alpha': 0.001, 'eta0': 0.1, 'learning_rate': 'optimal', 'penalty': 'l2'}]
Dynamic	Logistic Regression RFE	Logistic SGD	19	[{'alpha': 0.0001, 'eta0': 0.01, 'learning_rate': 'optimal', 'penalty': 'l1'}]
