# Supplementary material for: Angiogenic and immune predictors of neoadjuvant axitinib response in renal cell carcinoma with venous tumour thrombus
Source: Nat Commun. 2025 Apr 28;16:3870. doi: 10.1038/s41467-025-58436-8 (PMC12037771; doi:10.1038/s41467-025-58436-8)
Supplement: Supplementary file 3 — Reporting Summary [file 41467_2025_58436_MOESM3_ESM.pdf]

nature portfolio

Corresponding author(s): Dr James Jones

Last updated by author(s): Feb 13, 2025

Reporting Summary

Nature Portfolio wishes to improve the reproducibility of the work that we publish. This form provides structure for consistency and transparency in reporting. For further information on Nature Portfolio policies, see our Editorial Policies and the Editorial Policy Checklist.

Statistics

For all statistical analyses, confirm that the following items are present in the figure legend, table legend, main text, or Methods section.

n/a	Confirmed	
☐	☒	The exact sample size (*n*) for each experimental group/condition, given as a discrete number and unit of measurement
☐	☒	A statement on whether measurements were taken from distinct samples or whether the same sample was measured repeatedly
☐	☒	The statistical test(s) used AND whether they are one- or two-sided<br>*Only common tests should be described solely by name; describe more complex techniques in the Methods section.*
☐	☒	A description of all covariates tested
☐	☒	A description of any assumptions or corrections, such as tests of normality and adjustment for multiple comparisons
☐	☒	A full description of the statistical parameters including central tendency (e.g. means) or other basic estimates (e.g. regression coefficient) AND variation (e.g. standard deviation) or associated estimates of uncertainty (e.g. confidence intervals)
☐	☒	For null hypothesis testing, the test statistic (e.g. *F*, *t*, *r*) with confidence intervals, effect sizes, degrees of freedom and *P* value noted<br>*Give P values as exact values whenever suitable.*
☒	☐	For Bayesian analysis, information on the choice of priors and Markov chain Monte Carlo settings
☒	☐	For hierarchical and complex designs, identification of the appropriate level for tests and full reporting of outcomes
☐	☒	Estimates of effect sizes (e.g. Cohen's *d*, Pearson's *r*), indicating how they were calculated

Our web collection on statistics for biologists contains articles on many of the points above.

Software and code

Policy information about availability of computer code

Data collection	All software tools and version numbers are stated in the Methods:<br><br>Histology & Image analysis: HALO Software (Indica Labs) - HighPlex FL v3.1.0, Object Colocalization FL v1.0, Area Quantification FL v2.1.5, Area Quantification v2.4.3, Multiplex IHC v3.1.4.<br><br>Flow cytometry - FlowJo software.
Data analysis	All software tools and version numbers are stated in the Methods:<br><br>R - statistical and transcriptomic analysis:<br>- ggpubr (v0.6.0)<br>- Survival (v3.7.0)<br>- Survminer (v0.5.0)<br>- Cluster Profiler (v4.12.0)<br>- EnrichPlot (v1.24.0).<br><br>Python - machine learning models:<br>- scikit-learn (v1.4)

The GitHub link for our machine learning pipeline is available at https://github.com/CrispinLab/NAXIVA, as stated in the Code Availability statement.

For manuscripts utilizing custom algorithms or software that are central to the research but not yet described in published literature, software must be made available to editors and reviewers. We strongly encourage code deposition in a community repository (e.g. GitHub). See the Nature Portfolio guidelines for submitting code & software for further information.

Data

Policy information about availability of data

All manuscripts must include a data availability statement. This statement should provide the following information, where applicable:

- Accession codes, unique identifiers, or web links for publicly available datasets
- A description of any restrictions on data availability
- For clinical datasets or third party data, please ensure that the statement adheres to our policy

De-identified RNA-seq data have been deposited the Gene Expression Omnibus, accession GSE281304 [https://www.ncbi.nlm.nih.gov/geo/query/acc.cgi?acc=GSE281304]. The de-identified imputed, normalised dataset which was input to the ML models is provided in the Supplementary Information file. The data generated in this study are provided in the Source Data file.

Research involving human participants, their data, or biological material

Policy information about studies with human participants or human data. See also policy information about sex, gender (identity/presentation), and sexual orientation and race, ethnicity and racism.

Reporting on sex and gender	Included in the manuscript.
Reporting on race, ethnicity, or other socially relevant groupings	Not collected in this study
Population characteristics	Reported in the clinical paper, Stewart et al. British Journal of Cancer (2022)
Recruitment	Reported in the clinical paper, Stewart et al. British Journal of Cancer (2022)
Ethics oversight	Ethics approval for the NAXIVA trial was granted by the East of England—Cambridgeshire and Hertfordshire Research Ethics Committee (REC reference: 17/EE/0240).

Note that full information on the approval of the study protocol must also be provided in the manuscript.

Field-specific reporting

Please select the one below that is the best fit for your research. If you are not sure, read the appropriate sections before making your selection.

☒ Life sciences ☐ Behavioural & social sciences ☐ Ecological, evolutionary & environmental sciences

For a reference copy of the document with all sections, see nature.com/documents/nr-reporting-summary-flat.pdf

Life sciences study design

All studies must disclose on these points even when the disclosure is negative.

Sample size	The sample size was 20. Sample size was determined by the NAXIVA trial (PMID: 35739300).
Data exclusions	All evaluable patients were included.
Replication	The experimental findings come from a defined clinical trial population, so direct replication was not possible. External validation was carried out on public datasets as detailed in the manuscript.
Randomization	NAXIVA was a non-randomised phase II trial.
Blinding	As NAXIVA was a single-arm trial, no blinding was possible during data acquisition. To minimise bias whilst assessing the VTT length (i.e. outcome metric of the trial), this was performed by consensus by two central uroradiologists (PMID: 35739300).

Reporting for specific materials, systems and methods

We require information from authors about some types of materials, experimental systems and methods used in many studies. Here, indicate whether each material, system or method listed is relevant to your study. If you are not sure if a list item applies to your research, read the appropriate section before selecting a response.

Materials & experimental systems

n/a	Involved in the study
[ ]	[x] Antibodies
[x]	[ ] Eukaryotic cell lines
[x]	[ ] Palaeontology and archaeology
[x]	[ ] Animals and other organisms
[ ]	[x] Clinical data
[x]	[ ] Dual use research of concern
[x]	[ ] Plants

Methods

n/a	Involved in the study
[x]	[ ] ChIP-seq
[ ]	[x] Flow cytometry
[x]	[ ] MRI-based neuroimaging

Antibodies

Antibodies used	Details are provided in the Methods section of the manuscript: Immunohistochemistry was performed on the Leica Bond III platform by standard automated procedure. The following antibodies were used: CD8 (4B11 Leica PA0183), CD31 (JC70A Leica PA0414), Ki67 (MIB-1 Dako M7240). For immunofluorescence, 3-micron formalin-fixed paraffin-embedded (FFPE) sections were dewaxed in xylene and rehydrated in graded alcohols. Heat Induced Epitope Retrieval was performed in in Tris-EDTA pH9. After blocking, slides were incubated with primary antibodies at 4°C overnight. Antibodies used were as follows: CD31 (JC/70A, Abcam ab9498), CD34 (R&D Systems AF7227), SMA (Abcam ab5694), CD68 (KP1, Invitrogen MA5-13324), Ki67 (EPR3610, Abcam ab92742), CD8 (SP16, Invitrogen MA5-14548), Granzyme B (Leica NCL-L-GRAN-B), PD-1 (R&D Systems AF1086), CD4 (EPR6855, Abcam ab133616), FOXP3 (236A/E7, Abcam ab20034), CA9 (R&D Systems AF2188), CD3 (D7A6E, Cell Signalling Technology 85061S).
Validation	Manufacturer catalogue numbers are quoted in the Methods; validation details are available on the websites.

Clinical data

Policy information about clinical studies

All manuscripts should comply with the ICMJEguidelines for publication of clinical research and a completedCONSORT checklist must be included with all submissions.

Clinical trial registration	NCT03494816
Study protocol	Reported in the clinical paper, Stewart et al. British Journal of Cancer (2022)
Data collection	Reported in the clinical paper, Stewart et al. British Journal of Cancer (2022)
Outcomes	Reported in the clinical paper, Stewart et al. British Journal of Cancer (2022)

Plants

Seed stocks	*Report on the source of all seed stocks or other plant material used. If applicable, state the seed stock centre and catalogue number. If plant specimens were collected from the field, describe the collection location, date and sampling procedures.*
Novel plant genotypes	*Describe the methods by which all novel plant genotypes were produced. This includes those generated by transgenic approaches, gene editing, chemical/radiation-based mutagenesis and hybridization. For transgenic lines, describe the transformation method, the number of independent lines analyzed and the generation upon which experiments were performed. For gene-edited lines, describe the editor used, the endogenous sequence targeted for editing, the targeting guide RNA sequence (if applicable) and how the editor was applied.*
Authentication	*Describe any authentication procedures for each seed stock used or novel genotype generated. Describe any experiments used to assess the effect of a mutation and, where applicable, how potential secondary effects (e.g. second site T-DNA insertions, mosiacism, off-target gene editing) were examined.*

Flow Cytometry

Plots

Confirm that:

- [x] The axis labels state the marker and fluorochrome used (e.g. CD4-FITC).
- [x] The axis scales are clearly visible. Include numbers along axes only for bottom left plot of group (a 'group' is an analysis of identical markers).
- [ ] All plots are contour plots with outliers or pseudocolor plots.
- [x] A numerical value for number of cells or percentage (with statistics) is provided.

Methodology

Sample preparation	Included in the manuscript
Instrument	BD Symphony instrument
Software	FlowJo
Cell population abundance	Included in the manuscript
Gating strategy	Included in the manuscript

☒ Tick this box to confirm that a figure exemplifying the gating strategy is provided in the Supplementary Information.
